# The salience network is activated during self‐recognition from both first‐person and third‐person perspectives

**DOI:** 10.1002/hbm.26084

**Published:** 2022-09-21

**Authors:** Shoko Asakage, Tamami Nakano

**Affiliations:** ^1^ Graduate School of Frontiers Bioscience Osaka University Osaka Japan; ^2^ Graduate School of Medicine Osaka University Osaka Japan; ^3^ Center for Information and Neural Networks (CiNet) Osaka Japan

**Keywords:** first‐person perspective, precuneus, salience network, self‐recognition, third‐person perspective

## Abstract

We usually observe ourselves from two perspectives. One is the first‐person perspective, which we perceive directly with our own eyes, and the other is the third‐person perspective, which we observe ourselves in a mirror or a picture. However, whether the self‐recognition associated with these two perspectives has a common or separate neural basis remains unclear. To address this, we used functional magnetic resonance imaging to examine brain activity while participants viewed pretaped video clips of themselves and others engaged in meal preparation taken from first‐person and third‐person perspectives. We found that the first‐person behavioral videos of the participants and others induced greater activation in the premotor‐intraparietal region. In contrast, the third‐person behavioral videos induced greater activation in the default mode network compared with the first‐person videos. Regardless of the perspective, the videos of the participants induced greater activation in the salience network than the videos of others. On the other hand, the videos of others induced greater activation in the precuneus and lingual gyrus than the videos of the participants. These results suggest that the salience network is commonly involved in self‐recognition from both perspectives, even though the brain regions involved in action observation for the two perspectives are distinct.

## Graphical abstract

Salience network showed activation to video clips of the self from both first‐person and third‐person perspectives.

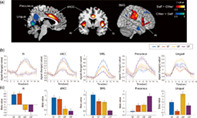

## 1 INTRODUCTION

We use mirrors and photographs to comprehend how our faces and bodies look from outside. Only humans, great apes, dolphins, and elephants can recognize their reflection in the mirror as themselves (Gallup, 1982). This third‐person perspective of one's appearance and behavior is relevant to one aspect of self‐consciousness that imagines how one is viewed by others (Fenigstein et al., 1975). We can also use our eyes to directly observe our body parts and actions. This first‐person visual image is integrated with other types of sensory (e.g., proprioception and vestibular) and motor information to produce elements of bodily self‐consciousness, such as the sense of body ownership and the agency of action (Blanke et al., 2015; Ehrsson et al., 2004; Haggard, 2017). Thus, recognition of ones' own appearance and behavior in both first‐person and third‐person perspectives is critical to the formation of our self‐consciousness. However, it remains unknown whether the neural basis of self‐recognition from these two perspectives is shared or distinct.

Neural correlates of self‐recognition from a third‐person perspective have been extensively studied using functional magnetic resonance imaging (fMRI) to examine brain activity in response to one's own face. Given that the human face is important for individual identification and holds social values (e.g., attractiveness), self‐face recognition from a third‐person perspective has important meaning for an individual. Several meta‐analyses of neuroimaging studies for self‐face recognition revealed that a broadly distributed brain network, including frontal, parietal, and occipital areas, is involved in self‐face recognition, with particularly strong involvement of the right hemisphere (Devue & Bredart, 2011; Hu et al., 2016; Platek et al., 2008; Sugiura, 2015). In particular, the anterior insular (AI) and anterior cingulate cortices (ACCs) as well as the inferior parietal lobes, which are the main areas that constitute the saliency network, consistently exhibited greater activation to one's own face than familiar and unfamiliar other faces (Devue et al., 2007; Morita et al., 2014; Ota & Nakano, 2021; Sugiura et al., 2000; Sugiura et al., 2006; Uddin et al., 2005). Self‐related activation in these brain regions disappeared or weakened by face deformation and the effects of depression and social anxiety (Kim et al., 2016; Ota & Nakano, 2021; Quevedo et al., 2018). Several previous studies also examined the brain activity in response to one's whole body and its movements (Devue et al., 2007; Sugiura et al., 2006). These studies found that the AI and ACC increased activity in response to one's own body as well as one's own face.

In terms of the first‐person perspective, previous neuroimaging studies have extensively examined brain activity that occurred while participants observed object‐related hand actions (Buccino et al., 2001; Caspers et al., 2010). They found that the neural circuits activated when an individual observed an action performed by another person were similar to those activated when that individual performed the same action. Furthermore, several studies found that some brain regions responded differently to the same action or body image depending on whether the frame of reference was egocentric or allocentric (Ge et al., 2018;Jackson et al., 2006; Saxe et al., 2006). For example, the first‐person body image induced greater activation in the sensory‐motor and extrastriate body area than the third‐person body image (Jackson et al., 2006; Saxe et al., 2006). However, these studies did not examine how brain activity differed when viewing one's own actions versus the actions of others from a first‐person perspective. Previous studies investigating the effect of perspective on autobiographical memory and scene perception have consistently reported that brain regions comprising the default mode network (DMN) show a greater increase in neural activity during the first‐person perspective than during the third‐person perspective (Viard et al., 2007; Vogeley et al., 2004; Young et al., 2013).

Previous neuroimaging studies have clearly demonstrated that taking both first‐person and third‐person perspectives is necessary for meta‐representations of the body and behavior because the self and various cortical regions are involved in this process (Vogeley & Fink, 2003). In particular, the salience network is primarily involved in perceiving one's own face and body from the third‐person perspective, whereas the sensory‐motor network and DMN are mainly involved in perceiving actions and scenes from the first‐person perspective. This raises the possibility that different brain networks are involved in self‐representation from different perspectives, even when observing the same behavior. However, given that meta‐representations of the self are formed by the integration of various perspectives, a common brain network may activate when an individual observes their own behavior, regardless of perspective. This study aimed to test which of these possibilities is correct.

Although the visual information contained in first‐person versus third‐person perspectives is completely different, the previous studies exclusively examined the effect of perspective by comparing brain responses to a single visual stimulus rotated 180 degrees (Jackson et al., 2006; Saxe et al., 2006). Visual information about an individual's self‐action observed via their own eyes is mainly based on parts of the body, such as hands. In contrast, visual information about an individual's self‐action as observed from third‐person perspective is based on the entire body, including the face and torso. To address these issues, we prepared different types of visual information about an action, that is, the action as observed from a first‐person perspective (movements of a hand) and that observed from a third‐person perspective (movements of the whole body including face), and compared brain activity for cases when the subject of the visual image was the participant versus another person for each perspective.

**FIGURE 1:**
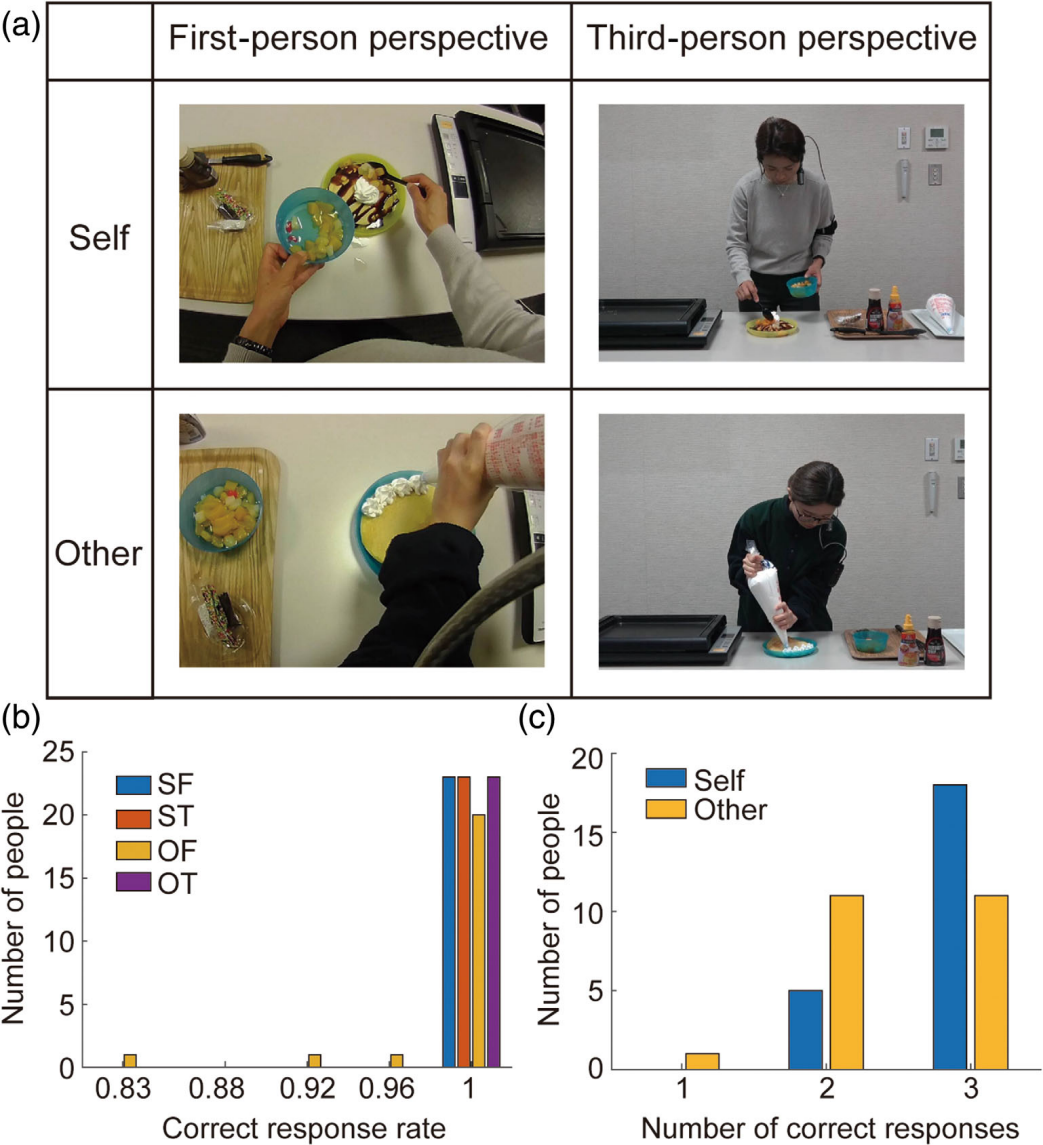
Experimental stimuli and behavioral results. (a) Video clips of the participant and other people preparing food, filmed from their own (first‐person) and third‐person perspectives. (b) Histogram of the correct response rate in self–other discrimination task. SF, ST, OF, and OT represent self‐images with a first‐person perspective, images of others with a first‐person perspective, self‐images with a third‐person perspective, and images of others with a third‐person perspective, respectively. (c) Histogram of the number of correct responses to the questions about meal preparation (three questions) for self and others' videos.

To this end, we created video clips that induced self‐recognition from first‐person and third‐person perspectives. To make it easier to distinguish between oneself and others in the first‐person perspective videos, we created stimuli by asking participants to perform daily natural actions from which we extracted similar action scenes. Specifically, the participants were instructed to prepare different meals in a kitchen. While they were preparing food, we simultaneously filmed their behavior using a camera fixed to their head (the first‐person perspective) and a camera placed in front of them (the third‐person perspective). Within 2 weeks of shooting, the participants were invited to undergo fMRI scanning while they watched the pretaped video clips of themselves and others engaged in meal preparation from the first‐person and third‐person perspectives (Figure 1). To enhance self–other discrimination for the video stimuli during the fMRI scans, the participants were told that they would be asked to answer questions about how they and the others cooked after each MRI session. Furthermore, we conducted a follow‐up experimental session to check that the participants were able to discriminate between themselves and the others in each video clip.

## 2 MATERIALS AND METHODS

### 2.1 Participants

A total of 23 college students participated in this study (mean age: 21.7 years, range: 20–24 years; 11 women). They had no abnormal neurological history and had normal vision that was either uncorrected or corrected by glasses. The review board of Osaka University approved the experimental protocol (FBS30‐4), and our procedures followed the guidelines outlined by the Declaration of Helsinki. All participants provided written informed consent prior to the experiment.

### 2.2 Stimuli and experimental procedure

Before the fMRI experiment, we shot several minutes of footage of each participant preparing a pizza, a parfait, and pancakes from two different perspectives simultaneously. The first‐person video of participant behavior was captured using a wearable camera attached to the participant's head (HX‐A100, Panasonic Corp.). The third‐person video of participant behavior was captured using a video camera placed in front of the meal preparation area (FDR‐AX55, Sony Marketing Inc.). The participants were instructed to employ their own methods to prepare the dishes using the available ingredients and tools located on the meal preparation table. During shooting, the participants wore their own clothes, including watches and accessories. These video clips were used for the “self” condition. We edited eight 5‐s video clips from each video (pizza, parfait, and pancakes) for each participant using video editing software (CyberLink PowerDirector 19, CyberLink Corp.). Thus, 24 video clips were prepared for each perspective. The footage from the two viewpoints was aligned so that the clips corresponded to the same period of meal preparation. The video images were 720 × 480 pixels.

For the “other” condition, we filmed videos of three men and three women engaged in the same meal preparation exercise from two different perspectives. Videos of individuals of the same sex as the participants were used for the “other” condition. Three actors were assigned to one of three meal preparations (pizza, parfait, or pancakes), and these actors did not participate in the fMRI experiment and were not acquainted with the participants of the fMRI experiment.

Because previous studies have reported that DMN activation associated with spontaneous self‐related mental activity tends to occur during rest intervals (Harrison et al., 2008; Raichle et al., 2001; Shulman et al., 1997), we presented a short video clip of a nonliving object during rest intervals to draw participants' attention toward the monitor. Specifically, we prepared twenty‐four 10‐s animated clips of nonliving objects taken from videos for babies (Baby Mozart, The Walt Disney Co.). These video images were 360 × 240 pixels.

During the fMRI experiment, the participants were asked to lie in an MRI scanner while wearing earplugs. Each participant's head was immobilized using a sponge cushion. They viewed visual stimuli on a screen (1280 × 720 pixels, refresh rate 30 Hz, and viewing angle = 27.1°) via a mirror placed in front of their eyes. All participants completed three sessions, and the meal preparation videos were shown in the same order (first session: parfait; second session: pizza; and third session: pancakes). In each session, we presented four types of meal preparation video clips (Figure 1), that is, video clips of the self from a first‐person perspective (SF), video clips of others from a first‐person perspective (OF), video clips of the self from a third‐person perspective (ST), and video clips of others from a third‐person perspective (OT), eight times each, in random order. There was a total of 24 trials for each condition (the total number of trials was 96). Between the meal preparation video clips, we showed short animated clips of nonliving objects for 10 s. In the “other” conditions (OF and OT), female participants were shown videos with female actors, male participants were shown videos with male actors, and the actors were different in each session. The stimulus presentation was controlled using Presentation (Neurobehavioral Systems Inc.). After each session, the participants were asked to respond to two “yes/no” questions about the meal preparation video clips to confirm that they had watched the video. The question for the first session was “did you/the other person put a Pocky in the parfait?” The question for the second session was “did you/the other person put an olive on the pizza?” The question for the third session was “did you/the other person use maple syrup on the pancakes?”

After the fMRI scanning day, we checked whether the participants were able to correctly distinguish themselves from the others in the videos for each perspective. To do this, we showed each participant the video clips on a PC laptop, presented in the same order as in the fMRI experiment. Immediately after viewing each video clip, the participants were asked to answer whether the image in the video clip was their own or someone else's.

Furthermore, the participants were asked to complete the Japanese version of the Self‐Consciousness Scale (SCS) questionnaire (Fenigstein et al., 1975; Sugawara, 1984), which comprises 11 and 10 items evaluating public and private self‐consciousness, respectively. We collected SCS scores because it contains several questions concerning self‐appearance, such as “I am concerned about my gestures and appearance” and “I am usually aware of my appearance.” Furthermore, a previous study reported a significant correlation between the SCS score and the extent of embarrassment to the self‐face (Morita et al., 2014). The participants rated each question using a 7‐point Likert scale. The score was calculated according to the SCS instructions.

### 2.3 Data acquisition

Functional images were acquired using multiband T2*‐weighted gradient echo‐planar imaging (EPI) sequences, which were obtained using a 3‐Tesla MRI scanner (MAGNETOM Vida, Siemens) with a 64‐channel array coil. We collected 500 scans per session (slice number = 45, slice thickness = 3 mm, repetition time [TR] = 1000 ms, echo time [TE] = 30 ms, flip angle = 60^°^, field of view [FOV] = 192 × 192 mm, voxel size [*x*, *y*, and *z*] = 3 × 3 × 3 mm, and multiband factor = 3). To generate anatomical reference images, we acquired a T1‐weighted structural image for each subject (magnetization prepared rapid gradient echo sequence, slice thickness = 1 mm, TR = 1900 ms, TE = 3.37 ms, flip angle = 9^°^, FOV = 256 × 256 mm, and voxel size [*x*, *y*, and *z*] = 1 × 1 × 1 mm). To correct the geometric distortion in the EPI, we also acquired field maps for each participant (Siemens standard double echo gradient echo field map sequence, slice thickness = 3 mm, TR = 753 ms, TE = 5.16 ms, flip angle = 90^°^, FOV = 192 × 192 mm, and voxel size [*x*, *y*, and *z*] = 3 × 3 × 3 mm).

### 2.4 Imaging data analysis

The acquired MRI data were processed using SPM12 and MATLAB R2020a. We discarded the first three EPI images in each session. To correct the image distortion caused by field inhomogeneity, we conducted field map correction using the SPM field map toolbox. We confirmed that head movement was <2 mm in all participants (22 of 23 participants moved their heads <1 mm), so we used all participants' data for the subsequent analysis. Then, the EPI images were realigned and unwarped. Each participant's structural image was coregistered to the mean of the motion‐corrected functional images. Subsequently, the EPI images were normalized to the standard brain template (Montréal Neurological Institute template) and smoothed using a Gaussian kernel filter with an 8‐mm full‐width‐at‐half‐maximum. After preprocessing, we conducted a voxel‐by‐voxel regression analysis of expected hemodynamic changes for the four conditions (SF/ST/OF/OT) using the general linear model on the single‐subject level. The trials in which the participants did not correctly discriminate between themselves and others in the video clips in the post hoc behavioral experiment were excluded from the subsequent fMRI analysis. We also regressed out global signal caused by motion artifacts using realignment parameters. On the group level, we performed a two‐way within‐subject analysis of variance (ANOVA) with factors for subject (self/other) and perspective (first‐person/third‐person). Post hoc analysis was conducted using paired *t*‐tests. For whole brain analyses, we used a family wise error (FWE) rate cluster‐corrected threshold of *p* < .05, with a cluster‐defining threshold of *p* < .001.

To compare the temporal dynamics of the blood oxygenation level dependence (BOLD) signal changes in the region of interest (ROI) between conditions, we extracted the signal intensity time course data in each ROI from each participant, applied a 128 Hz high‐pass filter, linearly interpolated the data at a resolution of 0.1 s, and converted it to *z*‐scores using the mean and variance. Then, we averaged the time course for each condition across trials from 2 s before to 15 s after the onset of the meal preparation video clip.

## 3 RESULTS

We first checked whether the participants were able to distinguish between themselves and others in each video clip captured from the first‐person and third‐person perspectives. The mean correct response rate was 1.0 for SF, ST, and OT conditions and 0.95 for OF condition (Figure 1b). These results confirmed that the participants automatically distinguished between themselves and others, regardless of the perspective. We also checked whether the participants attentively watched the video clips during MRI scanning. The mean correct response number for the three questions about meal preparation behavior for the self and other conditions was 2.78 (SD 0.42) and 2.43 (SD 0.59), respectively (Figure 1c). The mean score for the self‐condition was significantly higher than that for the other condition (paired *t*‐test, *t*_22_ = 2.91, *p* = .008).

**FIGURE 2:**
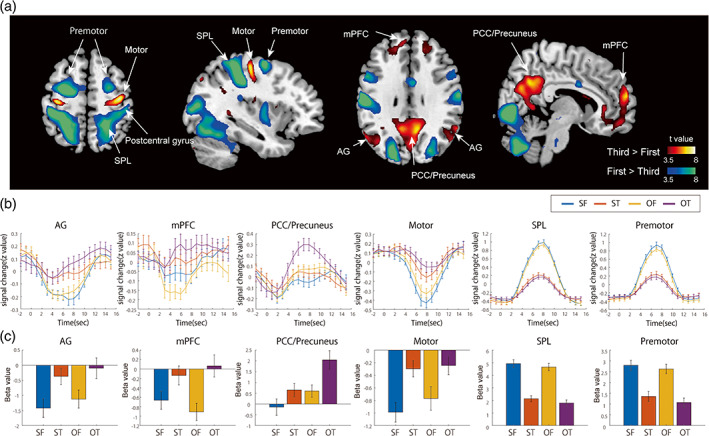
Brain regions exhibiting differences in activation between the first‐person and third‐person perspectives. (a) Brain regions showing greater activation when viewing the video clip taken from the third‐person perspective versus the first‐person perspective (hot colors, ST and OT > SF and OF), and the first‐person perspective versus the third‐person perspective (cool colors, SF and OF > ST and OT). The colored bars represent voxel‐level *t*‐values (FWEc *p* < .05, voxel level *p* < .001). (b) The mean time course of signal intensity in each ROI in response to each condition. AG, angular gyrus; mPFC, medial prefrontal cortex; PCC, posterior cingulate cortex; SPL, superior parietal lobe. (c) Mean beta value in each ROI for each condition. The error bars represent standard error. OF, video clips of others from a first‐person perspective; OT, video clips of others from a third‐person perspective; ROI, region of interest; SF, video clips of the self from a first‐person perspective; ST, video clips of the self from a third‐person perspective

Next, we analyzed brain activity evoked by the meal preparation video clips using a two‐way within‐subject ANOVA with self–other and perspective as factors. Significant main effects of face type and perspective were observed in the whole‐brain analysis (cluster‐level FWE [FWEc], *p* < .05; single voxel, *p* < .001). There was no significant interaction between the two factors. A direct comparison between the first‐person and third‐person perspectives using a paired *t*‐test revealed that the video images with the third‐person perspective induced greater activation in the medial prefrontal cortex (mPFC), precuneus/posterior cingulate cortex (PCC) gyrus, and bilateral angular gyrus (AG), which correspond to the DMN (Figure 2a, hot colors and Table 1; Huang et al., 2020; Raichle et al., 2001). Furthermore, we confirmed that these brain regions overlap with the locations of DMN nodes identified in a previous study using group ICA (Figure S1a). In contrast, the video images with the first‐person perspective induced greater activation in the premotor cortex, postcentral gyrus, superior parietal lobe (SPL), cerebellum, and visual areas (Figure 2a, cool colors). These brain areas exhibited a greatly increased BOLD signal in the SF and OF conditions compared with the ST and OT conditions, whereas the motor area, AG, and mPFC exhibited a significantly decreased BOLD signal for the SF and OF conditions compared with the ST and OT conditions (Figure 2b,c).

**TABLE 1:** Brain regions showing a significant difference in activation between the first‐person and third‐person perspectives

Brain regions	Cluster‐level	*k*	Laterality	MNI coordinates			Peak level *t*‐value
	*p*‐value			*x*	*y*	*z*	
(a) First > Third							
	<.0001	310,520					
Superior parietal lobule			R	22	−46	58	10.59
			L	−26	−50	56	12.12
Postcentral gyrus			R	40	−34	50	8.29
			L	−60	−14	28	13.02
Middle occipital gyrus			R	34	−78	12	13.24
			L	−24	−80	26	12.72
Fusiform gyrus			R	30	−50	−16	17.67
			L	−30	−52	−18	13.99
Brain stem			R/L	−6	−24	−8	8.2
Cerebellum			R	4	−62	−42	10.53
Premotor			L	−28	−6	60	11.57
Premotor	<.0001	611	R	26	−2	54	7.55
Posterior insula	<.0001	1146	L	−38	−6	2	7.56
Thalamus	<.0001	1321	L	−20	−26	2	10.47
Inferior frontal gyrus	.002	425	R	48	10	22	5.92
	<.0001	1146	L	−52	10	32	7.56
(b) First < Third							
	<.0001	141,890					
Posterior cingulate gyrus			R/L	−18	−44	−2	8.79
Precuneus			R/L	0	−68	26	7.3
Middle temporal gyrus			R	48	−42	6	9.29
			L	−58	−28	6	6.95
	<.0001	5903					
Medial prefrontal cortex			R/L	−14	50	10	7.2
Anterior cingulate gyrus			R/L	−10	36	10	5.53
Angular gyrus	.017		R	48	−66	38	5.67
	<.0001		L	−46	−70	40	6.88
Precentral gyrus	<.0001	917	R	38	−16	54	8.46
	.01	319	L	−36	−20	56	7.62
Cerebellum exterior	.046	216	R	16	−90	−32	5.12

*Note*: The cluster‐level statistics had a FEW‐corrected threshold *p* < .05. The *t‐*values represent voxel‐level uncorrected statistics (*p* < .001).

Abbreviation: MNI, Montreal Neurological Institute.

**FIGURE 3:**
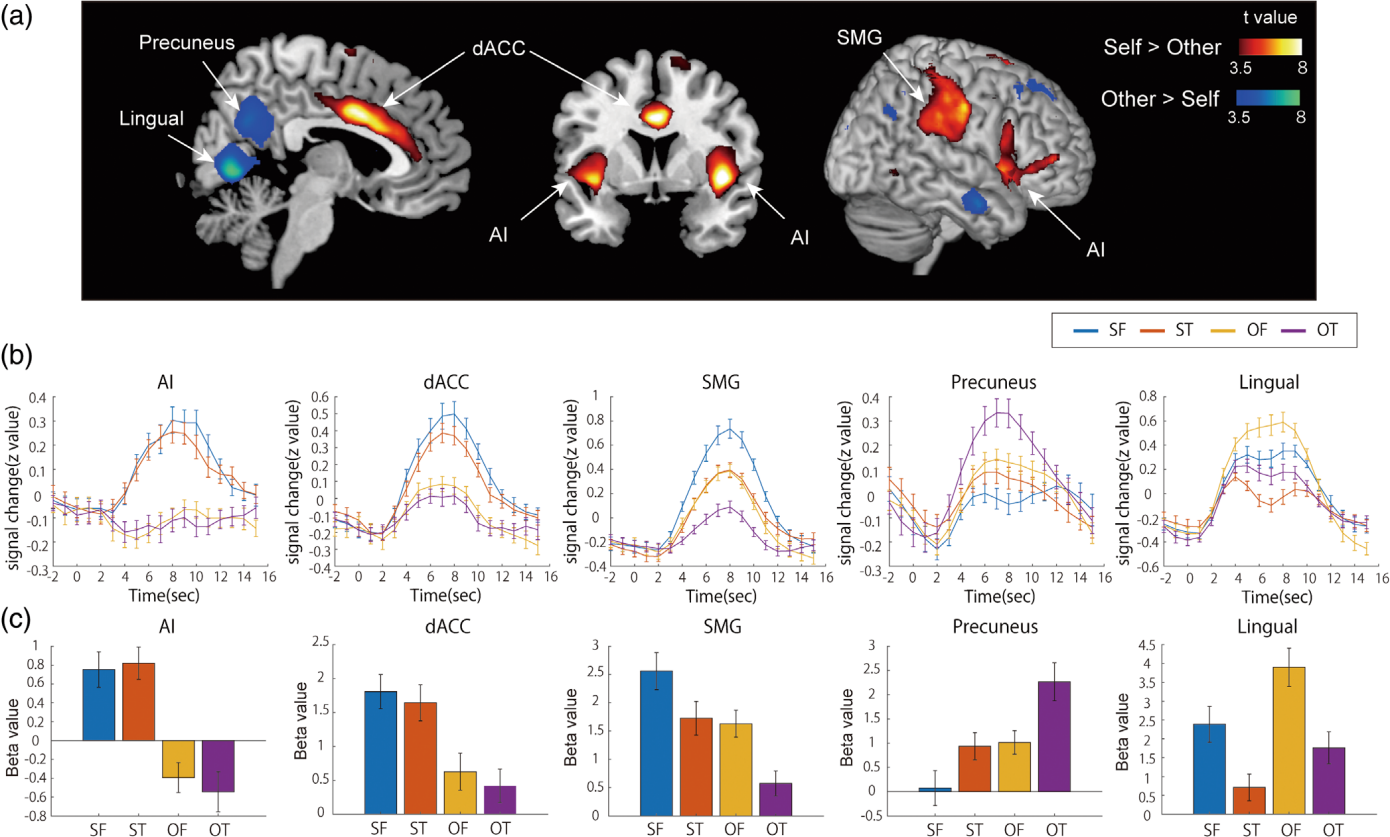
Brain regions exhibiting a difference in activation between video clips of the participant versus other individuals. (a) Brain regions showing greater activation when participants viewed the video clip of themselves versus others (hot colors, SF and ST > OF and OT), and of others versus themselves (cool colors, OF and OT > SF and ST). The colored bars represent voxel‐level *t*‐values (FWEc *p* < .05, voxel‐level *p* < .001). (b) Mean time courses of signal intensity in each ROI in response to each condition. AI, Anterior insular; dACC, dorsal anterior cingulate cortex; SMG, superior marginal gyrus. (c) Mean beta value in each ROI for each condition. The error bars represent standard error. FWE, family wise error; FWEc, cluster‐level FWE; OF, video clips of others from a first‐person perspective; OT, video clips of others from a third‐person perspective; ROI, region of interest; SF, video clips of the self from a first‐person perspective; ST, video clips of the self from a third‐person perspective

**FIGURE 4:**
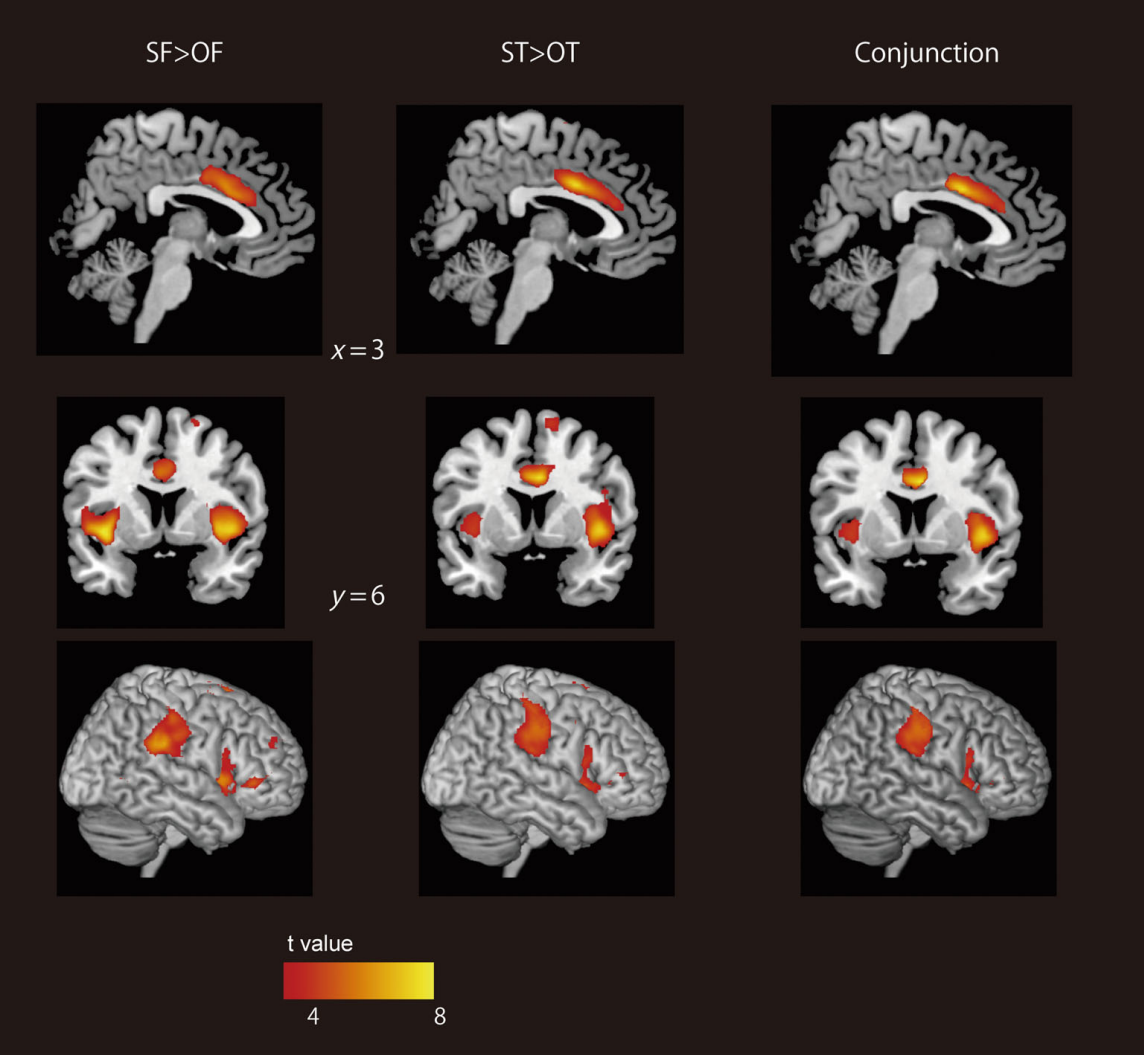
Brain regions showing greater activation when participants viewed images of themselves from each perspective. The left panels show the contrast for the first‐person perspective (SF > OF), and the center panels show the contrast for the third‐person perspective (ST > OT). The right panes show a result of conjunction analysis between the first‐person perspective (SF > OF) and the third‐person perspective (ST > OT). The colored bars represent voxel‐level *t*‐values (FWEc *p* < .05, voxel‐level *p* < .001). FWE, family wise error; FWEc, cluster‐level FWE; OF, video clips of others from a first‐person perspective; OT, video clips of others from a third‐person perspective; SF, video clips of the self from a first‐person perspective; ST, video clips of the self from a third‐person perspective.

Next, we compared the brain activity induced by video clips of the self versus others. As shown in Figure 3a (hot colors), the self‐image induced greater activation in the dorsal ACC (dACC), bilateral AI, and bilateral superior marginal gyrus (SMG; see Table 2). Previous neuroimaging studies using resting state analysis have indicated that these three regions compose the salience network (Huang et al., 2020; Krönke et al., 2020; Seeley et al., 2007; Touroutoglou et al., 2012). Furthermore, we confirmed that these brain regions overlap with the locations of salience network nodes identified in previous studies using group ICA as shown in Figure S1b (Huang et al., 2020; Krönke et al., 2020). Activation of the salience network when viewing the self‐image was consistently observed for both the first‐person and third‐person perspectives, even when analyzed separately (Figure 4a). The conjunction analysis of the first‐person and third‐person perspectives consistently revealed that the bilateral AI, dACC, and SMG showed greater activation in response to the self‐condition than to the other condition (Figure 4). The BOLD signal in the AI and dACC increased to the same extent in both the SF and ST conditions, but the BOLD signal in the SMG showed the greatest increase in the SF condition, followed by the ST and then the OF condition (Figure 3b,c). In contrast, the video clips of other people induced greater activation in the precuneus and lingual gyrus (Figure 3a, cool colors). The BOLD response in the precuneus was greatest for the OT condition, while that in the lingual gyrus was greatest for the OF condition (Figure 3b,c).

**TABLE 2:** Brain regions showing a significant difference in activation between the video images of the participant and other individuals

Brain regions	Cluster‐level	*k*	Laterality	MNI coordinates			Peak level *t*‐value
	*p*‐value			*x*	*y*	*z*	
(a) Self > Other							
Anterior insula	<.0001	2827	R	42	6	−4	9.51
	<.0001	1608	L	−40	6	−4	7.43
Supramarginal gyrus	<.0001	2541	R	46	−26	46	6.87
	<.0001	1368	L	−56	−28	38	7.00
Superior parietal lobule	<.0001	2541	R	34	−38	50	5.71
	<.0001	1368	L	−28	−42	68	4.68
Dorsal anterior cingulate gyrus	<.0001	2104	R/L	2	6	34	8.53
Inferior frontal gyrus	<.0001	2827	R	46	34	0	4.82
Superior frontal gyrus	.003	441	R	10	14	64	5.12
(b) Self < Other							
	<.0001	3381					
Lingual gyrus			R/L	8	−68	0	8.53
Precuneus			R/L	4	−60	30	5.44
Posterior cingulate gyrus			R/L	10	−48	30	5.16
Angular gyrus	.002	501	R	44	−58	30	4.30
Middle temporal gyrus	.049	234	R	60	−4	−22	5.46

*Note*: The cluster‐level statistics had an FWE‐corrected threshold *p* < .05. The *t‐*values represent voxel‐level uncorrected statistics (*p* < .001).

Abbreviation: FWE, family wise error; MNI, Montreal Neurological Institute.

We further analyzed the correlation between SCS scores and brain activity in terms of the contrast between the self and other condition. The mean score of the public SCS was 54.8 (SD 7.1, range 42–68) and that of the private SC was 48.6 (SD 6.3, range 37–63). No brain region showed a significant correlation between brain activity and SCS score for either the public or private SCS.

## 4 DISCUSSION

In this study, we examined brain activity in participants while they viewed videos of themselves and others from first‐person and third‐person perspectives. The first‐person videos of both the participants and other actors induced greater activation in the premotor area, postcentral gyrus, SPL, and cerebellum compared with the third‐person videos, whereas the third‐person videos induced greater activation in the DMN than the first‐person videos. For both perspectives, the salience network showed greater activation for video clips of the self than video clips of others, whereas the precuneus and lingual gyrus showed greater activation for video clips of others than video clips of the self. These results suggest that different brain regions are involved in the recognition of behaviors depending on the perspective, but the salience network is commonly involved in self‐recognition for both perspectives. On the other hand, contrary to our initial hypothesis, we found that the DMN was involved in the recognition of actions from the third‐person perspective, especially those of others.

Our data clearly indicate that the salience network is consistently involved in recognizing one's own image, whether the information is taken from the first‐person or third‐person perspective. The salience network is primarily composed of the AI and dACC, and sometimes includes the SMG (Huang et al., 2020; Krönke et al., 2020; Seeley et al., 2007). It appears to be involved in the processing of subjective salient stimuli related to the self (Seeley et al., 2007; Touroutoglou et al., 2012; Uddin, 2015). Previous neuroimaging studies have found that the dACC and AI are involved not only in visual self‐recognition (Jauk et al., 2017; Morita et al., 2014; Ota & Nakano, 2021), but also in the processing of a variety of self‐relevant internal information, such as interoceptive processing (Craig, 2009; Critchley et al., 2004), the perception of emotional and social pain (Eisenberger et al., 2003; Singer et al., 2004) and conflict monitoring (Cohen & Carter, 2004). The SMG was also found to be involved in self‐relevant processing, such as and the recognition of one's own face and emotions (Ota & Nakano, 2021; Silani et al., 2013). Moreover, these brain regions in the salience network also show an increase in neural activity in association with the sense of agency (Farrer & Frith, 2002; Ohata et al., 2020) and illusory body ownership (Ehrsson et al., 2004; Limanowski et al., 2014). These illusory feelings demonstrate that bodily self‐consciousness is formed by the multisensory integration of bodily stimuli, which include body‐related visual information (Blanke et al., 2015). A recent meta‐analysis proposed that self‐processing can be divided into three neural models: interoceptive processing, exteroceptive processing, and mental‐self processing (Qin et al., 2020). Neuroimaging studies showed that the AI is involved in all three types of processing (Scalabrini et al., 2021), the dACC is involved in interoceptive processing, and the Temporoparietal Junction, including the SMG, is involved in exteroceptive and mental self‐processing. Considering that the video clips of this study induced recognition of one's internal and external bodily state and behaviors, it is likely that all three types of self‐processing were involved and that the salience network, which comprises the AI, dACC, and SMG, was activated, regardless of perspective.

It is worth noting that only the ACC and AI have Von Economo neurons (VENs), which are large spindle‐shaped neurons with few dendrites (Allman, Tetreault, Hakeem, Manaye, et al., 2011). VENs are found only in animals that are capable of mirror self‐recognition, such as humans and great apes. VENs are overwhelmingly more abundant in the human brain than in the brains of great apes (Allman, Tetreault, Hakeem, & Park, 2011). In addition, chimpanzees who exhibit mirror self‐recognition had higher cortical thickness in the ACC than those who do not exhibit it (Hopkins et al., 2019). Given that the unique structure of VENs allows for rapid processing and transmission of information, the salience network may be involved in generating a unified feeling of “I am” across time by instantly integrating various self‐relevant information via these neurons. Notably, the temporal structure of spontaneous brain activity in adults with autism spectrum disorder (ASD) is abnormally tilted towards more powerful slow frequencies at the expense of faster frequencies in the salience network (Damiani et al., 2019). The anatomical study also revealed that the VEN in ASD fails to develop normally (Allman et al., 2005). Taken together, the altered temporal processing in the salience network may be related to weak self‐representations in patients with ASD (Uddin, 2011).

The past self‐images may have evoked autobiographical memories, resulting in distinct brain activity between the self and other conditions. In fact, the correct response rate of the memory task after the MRI scan was higher in the self‐condition than in the other condition. However, the hippocampus and precuneus, which are involved in the recall of autobiographical memory (Viard et al., 2007; Young et al., 2013), did not show higher neural activity during the self‐condition compared with the other condition. Given that the duration of video stimuli was short, we speculate that participants recognized themselves in the videos but did not necessarily recall past events related to them. Alternatively, the memory task after scanning could be completed by recalling information based on autobiographical memory, which may have led to a higher correct response rate in the self‐condition.

It could be argued that the salience network may have been activated by the feeling of embarrassment aroused by viewing self‐images. Indeed, because we cannot see our own faces directly, complex social emotions are evoked when we see ourselves from a third‐person perspective via mirrors and photographs. Previous studies reported that activity in the ACC and AI was correlated with perceived embarrassment when participants were asked to evaluate their own faces (Morita et al., 2014). In this study, because the participants were not instructed to evaluate their subjective feelings, we were not able to demonstrate whether activation in the salience network was induced by social emotions caused by self‐evaluation. However, the first‐person video clips of the self are less likely to evoke such social emotions, because the images are familiar and had actually been seen by participants before. Thus, the activation of the salience network by video clips of the self is unlikely to be explained by social emotion alone; rather, this activation appears to be related to viewpoint‐independent self‐recognition.

We also found that the first‐person videos of both the self and others activated the premotor, somatosensory, and posterior parietal areas, while deactivating the motor area. The video clips from the first‐person perspective mainly contained hand movements by which objects were manipulated from an egocentric framework. Consistently, previous neuroimaging studies have reported that viewing object‐directed hand movements induces activation in somatotopically organized premotor and parietal areas (Buccino et al., 2001; Jastorff et al., 2010). These findings demonstrate that observing actions from a first‐person perspective can recruit the same neural structures that are involved in the actual execution of the observed action, although actual execution is inhibited via suppression of activity in the motor cortex. This implies that actions observed from a first‐person perspective is understood by the stimulation of actual movement.

When compared with the first‐person perspective, the third‐person perspective of actions of both the self and others induced greater activation in the DMN in this study. It could be argued that activity in the DMN may have been less suppressed when seeing the third‐person video clips because the first‐person video clips required a greater attentional load to discriminate the self and other from hand movements alone. However, it is worth noting that the precuneus/PCC exhibited distinct activation when participants viewed the third‐person images (especially of others). This finding raises the possibility that the precuneus is actively involved in behavioral understanding from the third‐person perspective. Previous neuroimaging studies have consistently reported that the precuneus is involved in observing and imagining the actions of others from a third‐person perspective (Farrer & Frith, 2002; Petrini et al., 2014; Ruby & Decety, 2001; Vogeley et al., 2004). Given that the video clips captured from the third‐person perspective showed holistic movements of the whole body, including the face, in the surrounding environment, our findings suggest that the precuneus is involved in the objective and holistic understanding of the actions of others.

Many previous studies have shown that the DMN is involved in self‐relevant information processing (Northoff & Bermpohl, 2004). For instance, the mPFC and precuneus/PCC showed activation during a self‐reflection task in which participants expressed ideas about their personal traits, attitudes, values, and relationships in a social context (D'Argembeau et al., 2012; Johnson et al., 2002; Kim & Johnson, 2012; Peer et al., 2015). Such tasks generally require subjects to intentionally describe themselves in words. A meta‐analysis comparing brain activity between nonverbal and verbal self‐relevant processing revealed that verbal self‐relevant processing activated the DMN, whereas nonverbal self‐relevant processing activated the salience network (Frewen et al., 2020; Uddin et al., 2007). Taken together, with the present finding of the DMN exhibiting greater activation in response to the video clips from the third‐person perspective than to those from the first‐person perspective, it is likely that the salience network is involved in automatic and subjective representations of the self, whereas the DMN is involved in more intentional and objective representations of the self.

Correspondingly, previous studies have reported that the temporal characteristics of resting‐state activity in cortical midline structures, including the mPFC, dACC, and PCC, are positively correlated with the private SCS score (Huang et al., 2016; Wolff et al., 2019), while the gray matter density in the precuneus/PCC is positively correlated with the public SCS score (Morita et al., 2021). In contrast, both private and public SCS scores showed no correlations with the brain activity that occurred during self–other distinction in this study. This difference may have occurred because we analyzed the correlation between brain activity during the task and the SCS score and not brain activity at rest, as was analyzed in previous studies.

## 5 CONCLUSIONS

Numerous studies have shown that the major brain networks, the salience network, and DMN, are involved in processing various types of self‐relevant information (Araujo et al., 2015; Craig, 2009; Frewen et al., 2020; Northoff & Bermpohl, 2004; Uddin et al., 2007). This study provides new insights regarding the functions of the salience network. Particularly, our data indicate that it is involved in visual self‐recognition from both the first‐person and third‐person perspectives, despite the differences in the self‐image information obtained. This suggests that the salience network is involved in generating unified representation of the self‐regardless of perspective. Further, this study revealed that the DMN is activated by observing third‐person actions, and that the precuneus responds more strongly to third‐person images of others than to those of the self. This suggests that the DMN is involved in a holistic and objective understanding of human behavior, especially the behavior of others.

## AUTHOR CONTRIBUTIONS

Tamami Nakano designed experiments. Shoko Asakage and Tamami Nakano created, collected, and analyzed data. Shoko Asakage and Tamami Nakano wrote the article.

## CONFLICT OF INTEREST

The author declares that there is no conflict of interest that could be perceived as prejudicing the impartiality of the research reported.

## ETHIC STATEMENTS

The review board of Osaka University approved the experimental protocol (FBS30‐4).

All procedures in this study were conducting following this protocol and the guidelines outlined by the Declaration of Helsinki.

## INFORMED CONSENT

All participants provided written informed consent prior to the experiment.

## Supporting information


**FIGURE S1** Comparison of brain activation map with locations of DMN and Salience Network nodes. (a) Overlap of brain activation map for third > first contrast with locations of four DMN nodes (green circle) identified in a previous study (Kronke et al., 2020). (b) Overlap of brain activation map for self > other contrast with locations of five Salience Network nodes identified in previous studies (green circle: Kronke et al., 2020; blue circle: Huang et al., 2020). DMN, default mode network.

## Data Availability

The data that support the findings of this study are available on request from the corresponding author Tamami Nakako.
