# Supplementary material for: The salience network is activated during self‐recognition from both first‐person and third‐person perspectives
Source: Hum Brain Mapp. 2022 Sep 21;44(2):559–70. doi: 10.1002/hbm.26084 (PMC9842878; doi:10.1002/hbm.26084)

Supplementary Figure 1

Comparison of brain activation map with locations of DMN and Salience Network nodes. (A) Overlap of brain activation map for third > first contrast with locations of four DMN nodes (green circle) identified in a previous study (Kronke et al., 2020). (B) Overlap of brain activation map for self > other contrast with locations of five Salience Network nodes identified in previous studies (green circle: Kronke et al., 2020; blue circle: Huang et al., 2020).

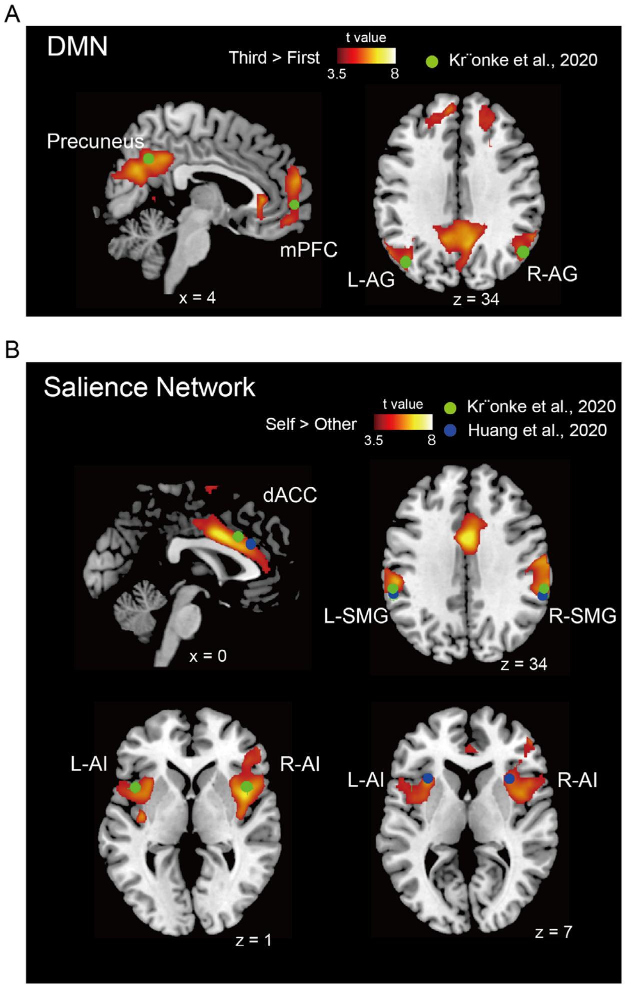

Supplement: Supplementary file 1 — FIGURE S1 Comparison of brain activation map with locations of DMN and Salience Network nodes. (a) Overlap of brain activation map for third > first contrast with locations of four DMN nodes (green circle) identified in a previous study (Kronke et al., 2020). (b) Overlap of brain activation map for self > other contrast with locations of five Salience Network nodes identified in previous studies (green circle: Kronke et al., 2020; blue circle: Huang et al., 2020). DMN, default mode network. [file HBM-44-559-s001.pdf]
